# Secreted frizzled‐related protein 2 promotes the osteo/odontogenic differentiation and paracrine potentials of stem cells from apical papilla under inflammation and hypoxia conditions

**DOI:** 10.1111/cpr.12694

**Published:** 2019-09-30

**Authors:** Haoqing Yang, Guoqing Li, Nannan Han, Xiuli Zhang, Yangyang Cao, Yu Cao, Zhipeng Fan

**Affiliations:** ^1^ Laboratory of Molecular Signaling and Stem Cells Therapy Beijing Key Laboratory of Tooth Regeneration and Function Reconstruction Capital Medical University School of Stomatology Beijing China; ^2^ Molecular Laboratory for Gene Therapy and Tooth Regeneration Beijing Key Laboratory of Tooth Regeneration and Function Reconstruction Capital Medical University School of Stomatology Beijing China; ^3^ Department of Periodontology Capital Medical University School of Stomatology Beijing China; ^4^ Department of General Dentistry Capital Medical University School of Stomatology Beijing China

**Keywords:** hypoxia, inflammation, osteo/odontogenic differentiation, paracrine, SFRP2, stem cells from apical papilla

## Abstract

**Objectives:**

Mesenchymal stem cell (MSC)‐based dental tissue regeneration is a potential treatment method in future, while inflammation and hypoxia niche will affect MSC‐mediated tissue regeneration. In this research, we intended to investigate the influence and mechanism of secreted frizzled‐related protein 2(SFRP2) on MSC function under inflammation and hypoxia conditions.

**Material and methods:**

Stem cells from apical papilla (SCAPs) were used in this study. The alkaline phosphatase (ALP) activity, Alizarin Red S staining, scratch‐simulated wound migration and transwell chemotaxis assay were used to evaluate the functions of SFRP2. The Western blot, real‐time RT‐PCR and ChIP assays were used to evaluate the mechanism of SFRP2.

**Results:**

Under inflammation and hypoxia conditions, the over‐expression of SFRP2 could enhance the osteo/odontogenic differentiation ability. Mechanismly, SFRP2 inhibited canonical Wnt/β‐catenin signalling pathway and then inhibited the target genes of nuclear factor kappa B (NFkB) signalling pathway. Inflammation or hypoxia conditions could promote the expression of lysine demethylase 2A (KDM2A) and repress SFRP2 transcription through decreasing histone methylation in the SFRP2 promoter. Besides, proteomic analysis showed that SFRP2 promoted SCAPs to secret more functional cytokines, which improve the migration, chemotaxis and osteo/odontogenic ability of MSCs.

**Conclusions:**

Our discoveries revealed that SFRP2 enhanced the osteo/odontogenic differentiation and paracrine potentials of SCAPs under hypoxia and inflammation conditions and provided a potential cytokine for promoting tissue regeneration in hypoxia and inflammatory niche.

## 1 INTRODUCTION

Pulpitis and periapical periodontitis are the most prevalent oral diseases, and root canal treatment is the primary therapy. However, teeth can become susceptible to fracture, leading to a higher incidence of extraction.^1^, ^2^ These years, regenerative endodontic procedures became an expanding field that aims for regeneration of a healthy and functional dentin‐pulp complex (DPC) that is capable of forming new dentin.^3^, ^4^ This procedure consists of chemical debridement, disinfection of the root canal and evoked bleeding after instrumentation beyond the apex; thus, stem cells could be delivered from the apical region to the whole extent of the root canal to regenerate the DPC.^5^, ^6^

So, development of mesenchymal stem cell (MSC)‐based regenerative endodontic procedures has made it possible for more ideal dental pulp tissue engineering. MSCs were first isolated from the tissue of bone marrow and are capable of self‐renewal and multiple differentiation. MSCs from other tissues were also found, such as adipose tissue‐derived stromal cells (ADSCs), periodontal ligament stem cells (PDLSCs), stem cells from the apical papilla (SCAPs), and dental pulp stem cells (DPSCs).^7^, ^8^, ^9^ These cells not only have the potential of MSCs and are capable of generating mineralized tissues.^10^, ^11^ Studies have proved the advanced ability of multiple MSCs derived from dental tissues in regenerating DPC.^12^, ^13^, ^14^ Among those dental tissue‐derived MSCs, SCAPs, isolated from apical papilla of immatured teeth root, showed higher proliferation rate and increasingly stronger osteo/odontogenic potential than PDLSCs and DPSCs.^15^, ^16^ And the differentiation capacity made SCAPs as a promising alterative seed cell for MSC‐based tissue regeneration.^17^

Except for the characteristics of MSCs, microenvironmental niche, which supports and maintains the functions of MSCs, is also an important factor for determining MSCs behaviour and tissue regeneration. Hypoxia is a common microenvironmental niche. In normal tissues of human adult, oxygen levels range from 2% to 9%.^18^ As previously studies showed oxygen levels in rabbit or rat incisor pulp were 3%~4.7%.^19^, ^20^ At present, MSCs are mainly isolated and cultured under normoxia (20%‐21% O_2_) in vitro, which is obviously different from their in vivo environment. Interestingly, studies have shown the opposite result about whether hypoxic conditions inhibit the osteo/odontogenic differentiation of MSCs derived from dental tissues. Some studies showed that changed cells characteristics and decreased osteoblast formation and mineralization were found in hypoxia condition.^21^, ^22^ However, other studies showed that hypoxia condition could up‐regulate osteogenic‐related genes in MSCs derived from dental tissues.^23^, ^24^ In addition, the dental pulp can become infected due to pulpitis. This leads to an inflammatory environment in the root canal, which will impair the MSCs function and weaken the osteo/odontogenic differentiation.^25^, ^26^ Altogether, the clinic inflammation and hypoxia niche in root canal will impair the MSC function and DPC regeneration, while the mechanism is still unclear.

Wnts are a family of secreted proteins that plays important role in skeletal development, embryogenesis and organogenesis. Important role of Wnt/β‐catenin signalling on formation and maintenance of bone and teeth has been defined.^27^ As endogenous Wnt regulators, secreted frizzled‐related protein (Sfrps) family contains five SFRP members, which are extracellular inhibitors of Wnt signalling that play important roles in both embryogenesis and oncogenesis.^28^, ^29^ And SFRP2 is a secreted protein produced by MSCs, which plays an important role in enhancing anti‐apoptosis ability of MSCs and self‐renewal under hypoxia condition.^30^, ^31^ In previous studies, we demonstrated that SFRP2 could increase the ability of osteo/odontogenic differentiation in MSCs, and SFRP2 was a target gene of lysine demethylase 2A (KDM2A).^32^ While under hypoxia and inflammation conditions, the role and mechanism of SFRP2 on MSC function are still uncertain.

In present search, we use SCAPs and intrigued to explore the function and underlying mechanisms of SFRP2 on MSCs under inflammation and hypoxia conditions. Our results revealed that SFRP2 could promote the osteo/odontogenic differentiation and paracrine potentials of SCAPs. Our discoveries provided new insights into the underlying mechanism of MSCs in microenvironmental niche and potential target for clinical applications.

## 2 MATERIALS AND METHODS

### 2.1 Cell cultures

All tooth tissues were obtained with informed patient agreement and under approved guidelines set by the Beijing Stomatological Hospital, Capital Medical University. Teeth were first disinfected with 75% ethanol and then washed with phosphate‐buffered saline (PBS). Periodontal ligament and apical papilla were separated gently from the middle one‐third of the teeth root or the tip of the unmatured tooth, respectively. Human BMSCs were obtained from ScienCell Research Laboratories. The culture of MSCs was described in our previous study.^32^ Cells at passage 3‐5 were used in subsequent experiments.

To induce osteo/odontogenic differentiation, we seeded 2.0 × 10^5^ cells into each well of six‐well plates. When cells reached 80% confluence, we changed the medium to the StemPro^®^ Osteogenesis Differentiation Medium (Invitrogen) for up to 14 days.

For hypoxia condition, cells were cultured under the condition by using a humidified incubator in a mixture of 92% N_2_, 5% CO_2_ and 3% O_2_ at 37°C. To mimic inflammation condition, the cells were stimulated with 10 ng/mL TNFα for indicated times.

To inhibit the WNT signalling pathway, the cells were treated with 10 μmol/L IWR‐1‐endo (APEXBIO) for 2 days.

### 2.2 Immunohistochemistry staining

The healthy pulp of human impacted third molar teeth and pulpitis tissue removed for acute pulpitis were immersed in formalin for 48 hours, then imbedded in paraffin, and sliced into 5 μm sections. For immunohistochemistry staining, the pulp tissue sections were deparaffinized and treated with antigen retrieval and then incubated in 3% H_2_O_2_ for 10 minutes. Goat serum was used to block non‐specific antibody binding. Then, sections were incubated with a primary polyclonal antibody against SFRP2 (Cat No. 06‐004, Millipore) at 4°C overnight. Then, horseradish peroxidase‐conjugated anti‐rabbit secondary antibody (Promega Madison) and detection reagents were used.

### 2.3 Plasmid construction and viral infection

Using gene synthesis and restriction enzymes, we constructed the plasmids and identified the sequence.^32^ Hemagglutinin (HA) tag was merged with human full‐length SFRP2 cDNA, and they were sub‐cloned into the pQCXIN retroviral vector with AgeI and NotI restriction sites. Short hairpin RNAs (shRNAs) with complementary sequences of target genes, including SFRP2, BCOR and KDM2A, were sub‐cloned into the pLKO.1 lentiviral vector (Addgene). Scramble shRNAs (Scramsh) were purchased from Addgene. The target sequence for the shRNAs is as follows: SFRP2 shRNA (SFRP2sh), 5′‐ttgatgtaggttatctccttc‐3′; BCOR shRNA (BCORsh), 5′‐gatggcttcagtgctatat‐3′; KDM2A shRNA (KDM2Ash), 5′‐tttccaagccaatggtttc‐3′.

### 2.4 Reverse transcriptase polymerase chain reaction and real‐time RT‐PCR

We isolated total RNA from cells with Trizol reagent (Invitrogen). The protocol of RT‐PCR and real‐time PCR reactions was depicted in our previous work.^32^ The primers for specific genes were displayed in Table S1.

### 2.5 Western blot analysis

Total protein was obtained from cells after lysed in RIPA buffer. The Western blot was performed as described in our previous work.^32^ The information of primary antibodies was HA (Cat No. 3724, Cell Signaling Technology), SFRP2 (Cat No. 06‐004, millipore), KDM2A (Cat No. ab31739, abcam), BCOR (Cat No. 12107‐1‐AP, proteintech), phosphorylated β‐catenin (Cat No.2009, Cell Signaling Technology), GAPDH (Cat No. C1312, Applygen) and β‐actin (Cat No. C1313, Applygen).

### 2.6 Alkaline phosphatase activity assay and alizarin red staining

For ALP activity assay, cells were cultured with osteogenesis differentiation medium in 6‐well plate for 5 days, and ALP activity assay was performed as described in our previous work.^32^ For Alizarin Red staining, cells were cultured in osteogenesis differentiation medium for 2 weeks according to the manufacturer's suggested protocol, as described in our previous study.^32^

### 2.7 Chromatin immunoprecipitation assays

We used a ChIP assay kit (Merck Millipore) according to the manufacturer's protocol. About 2.0 × 10^6^ cells were used in each ChIP experiment. ChIP assay was described in previous article.^32^ The DNA samples were detected by using real‐time PCR analysis. To amplify the KDM2A binding site in the SFRP2 promoter, we designed primers with the following sequences: forward, 5′‐cgtatgccatgtaaagttctgctcatacg‐3′; and reverse, 5′‐ gttcagcagcctgtcggtgt‐3′.

### 2.8 Preparation of conditional medium

SCAPs‐Vector and SCAPs‐HA‐SFRP2 cells were cultured and expanded in serum‐containing complete medium under normoxic conditions in 100 mm tissue culture dishes. When cells reached ∼80% confluence, the medium was changed to serum‐free α‐MEM (15 mL) medium and cultured for another 72 hours under hypoxic condition. Next, the supernatant of SCAPs‐Vector cells (Vector‐CM) and SCAPs‐HA‐SFRP2 cells (SFRP2‐CM), was collected, centrifuged at 13 000 × g at 4°C for 10 minutes, and stored at −80°C before use.

### 2.9 Sample preparation and proteomic analysis

Then, the culture supernatants of SCAPs‐Vector and SCAPs‐HA‐SFRP2 were collected (n = 3) for protein extraction, identification and quantification. The proteins were digested in trypsin (Promega) at 37°C overnight. Three iTRAQ Reagent 4‐plex kits (AB Sciex Inc) were used to label peptide samples with iTRAQ. The labelled peptide fragments from each sample were with an RP analytical column (Durashell‐C18, 4.6 mm × 250 mm, 5 μm, 100 Å) at a flow rate of 700 µL/min. Redissolved each peptide fragments in 2% methyl alcohol and 0.1% formic acid, then centrifuged at 16000 ***g*** for 10 minutes. The LC‐MS/MS was carried out by using Easy‐nLC nanoflow HPLC system which is connected to Q Exactive mass spectrometer (Thermo Fisher Scientific). The raw data were analysed with the Proteome Discoverer 1.4 software (Thermo Fisher Scientific) to identify the proteins.

### 2.10 Scratch migration assay

The 2.0 × 10^5^ PDLSCs, SCAPs or BMSCs were seeded onto each well of six‐well plate. Cross‐scratch with a 200 μL pipette tip in the cell layer along the diameter of the well, then half routine medium were exchanged with the Vector‐CM and SFRP2‐CM and captured images at the time point of 0, 24 and 48 hours. Image‐Pro 1.49v (National Institutes of Health) was used to analyse the data.

### 2.11 Transwell chemotaxis assays

MSCs were cultured in the transwell chambers which have an 8 μm pore size membrane (Corning Costar). BMSCs or PDLSCs (2.0 × 10^4^ cells) were seeded in the upper chamber with 100 μL serum‐free medium. 300 μL routine medium with 300 μL Vector‐CM or SFRP2‐CM was used in the bottom chamber. After 24 hours, we counted the transferred cell numbers in randomly selected fields using microscope (OLYMPUS) at 200× magnification.

### 2.12 CFSE assays

Cells were stained according to the CellTrace™ certified functional safety expert (CFSE) cell proliferation kit protocol (Invitrogen) for labelling cells in suspension and were then seeded at a density of 5.0 × 10^4^ cells/plate on 6‐well plates. Cells were harvested with 0.25% trypsin after 6 days of culture and analysed using a flow cytometer (Calibur; BD Biosciences) with 488 nm excitation and emission filters appropriate for fluorescein. The proliferation index was calculated by Modfit LT program.

### 2.13 Statistical analysis

All statistical calculations were performed using SPSS10 statistical software. Statistical significance was determined using one‐way ANOVA or Student's *t* test, with *P* values <.05 being considered significant.

## 3 RESULTS

### 3.1 SFRP2 enhanced the osteo/odontogenic differentiation potential of SCAPs under the hypoxia condition

Firstly, we investigate the SFRP2 expression in pulpitis, and the real‐time RT‐PCR and immunohistochemistry results showed that the expression of SFRP2 was decreased in pulpitis tissues compared with that in normal pulp tissues (Figure S1).

**Figure 1:**
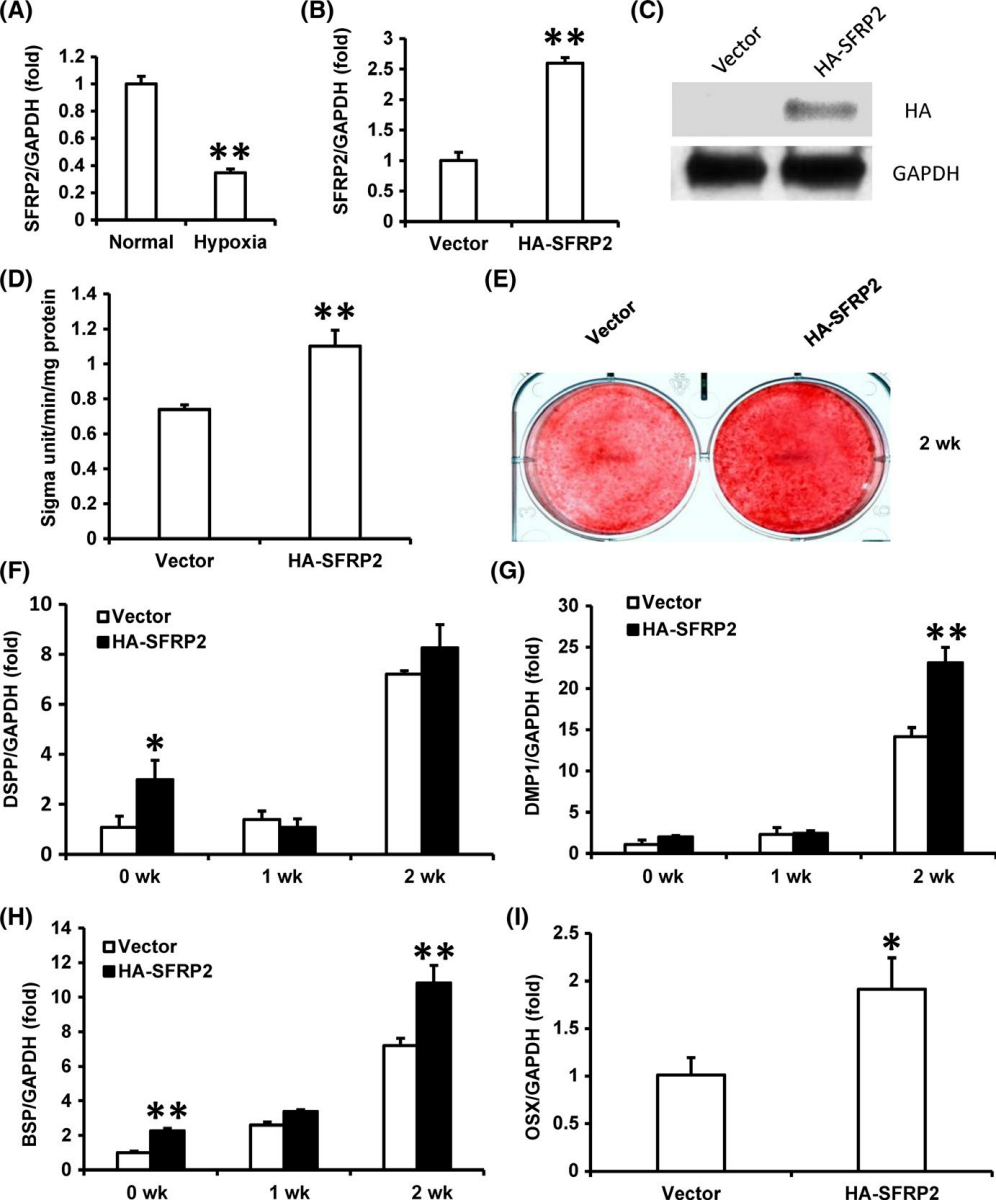
Over‐expression of SFRP2 promoted osteo/odontogenic differentiation potential of SCAPs under the hypoxia condition. SCAPs were cultured under 3% hypoxia condition. A, Real‐time RT‐PCR result showed expression level of SFRP2 at 24 h under hypoxia condition. B,C, Real‐time RT‐PCR and Western blot assays confirmed the expression of SFRP2 in SCAPs. D, ALP activity at 5 d after induction. E, Alizarin Red staining results at 2 wk after induction. F‐I, Real‐time RT‐PCR displayed expressions of DSPP (F), DMP1 (G), BSP (H) and OSX (I). GAPDH was used as internal control in Real‐time RT‐PCR.GAPDH was used as internal control in Western blot. Student's *t* test was performed to determine statistical significance. All error bars represent the SD (n = 3). *P ≤ .05; **P ≤ .01

To investigate whether hypoxia influenced the function of SFRP2, we compared SCAPs cultured in normal culture condition (normoxia) and hypoxia condition (3% O_2_). After 24 hours, the expression of SFRP2 was significantly decreased in the hypoxia group compared with normoxia group (Figure 1A). Next, we ectopically over‐expressed SFRP2 in SCAPs by infection with a retroviral construct expressing SFRP2. After selection by using 600 µg/mL G418 for 10 days, real‐time RT‐PCR and Western blot verified the over‐expression efficiency (Figure 1B, 1C). Then, the cells were cultured in osteogenic‐inducing medium under hypoxia conditions. Five days after the induction, increased ALP activity was found in SCAPs‐HA‐SFRP2 compared with control group (SCAPs‐Vector) (Figure 1D). After 2 weeks of induction, Alizarin Red staining results revealed remarkably improvement of mineralization in SCAPs‐HA‐SFRP2 than that of SCAPs‐Vector (Figure 1E). Real‐time RT‐PCR results displayed the increasing expression of osteo/odontogenic‐related genes, including dentin sialophosphoprotein (DSPP) at 0 week, dentin matrix acidic phosphoprotein 1 (DMP1) at 2 weeks, and bone sialoprotein (BSP) at 0 and 2 weeks in SCAPs‐HA‐SFRP2 compared with control group (Figure 1F‐H). Furthermore, real‐time RT‐PCR result displayed that the expression of OSX, the key transcription factor, was also up‐regulated in SCAPs‐HA‐SFRP2 compared with control group (Figure 1I).

Next, we detected the effect of SFRP2 on cell migration and proliferation in a hypoxic environment. The scratch migration assays showed that over‐expression of SFRP2 promoted the migration ability in SCAPs compared with control group at 24 hours and 48 hours in hypoxia condition (Figure S2A,B). Furthermore, CFSE assay results showed that over‐expression of SFRP2 enhanced the cell proliferation of SCAPs compared with control group in hypoxia condition (Figure S2C‐E). We wondered whether SFRP2 still have this effect under the switch from hypoxia to normal conditions. So, the SCAPs were treated with 3% O_2_ for 48 hours and then put them into the condition of 21% O_2_. The scratch migration assays showed that SFRP2 promoted the migration ability in SCAPs compared with control group at 48 hours (Figure S3A,B), while the CFSE assay results showed that there was no difference of cell proliferation between SCAPs‐HA‐SFRP2 and SCAPs‐Vector under the switch from hypoxia to normal conditions (Figure S3C‐E).

### 3.2 SFRP2 promoted the osteo/odontogenic differentiation in SCAPs under the hypoxia and inflammation conditions

**Figure 2:**
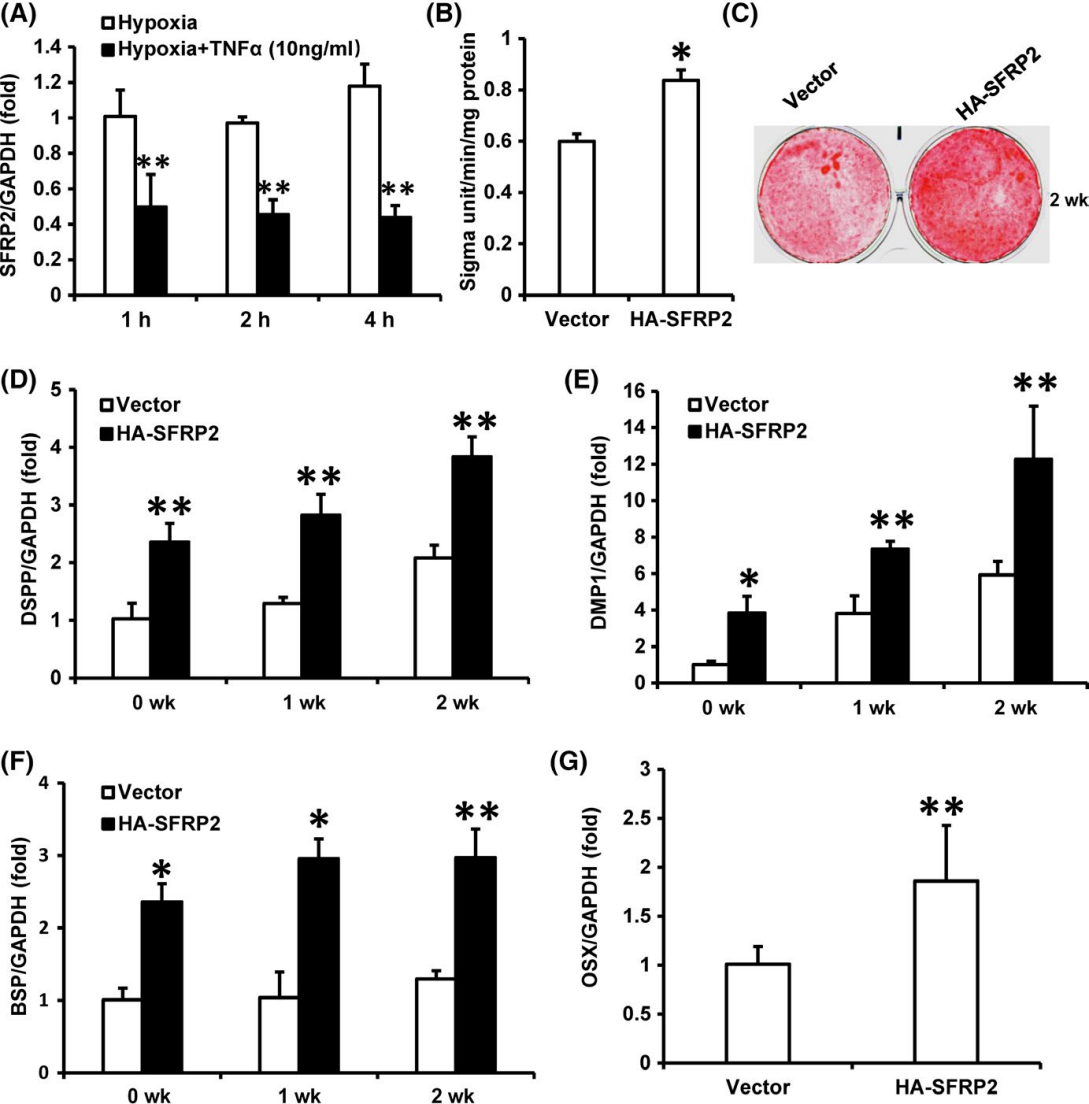
SFRP2 over‐expression enhanced the osteo/odontogenic differentiation in SCAPs under hypoxia condition with TNFα simulation. SCAPs were cultured under 3% hypoxia condition with 10 ng/ml TNFα stimulation. A, Real‐time RT‐PCR showed decreased SFRP2 expression in hypoxia with 10 ng/mL TNFα treatment. B, ALP activity at 5 d after induction. C, Alizarin Red staining results at 2 wk after induction. D‐G, Real‐time RT‐PCR results displayed expression levels of DSPP (D), DMP1 (E), BSP (F) and OSX (G). GAPDH was used as internal control. Student's *t* test was performed to determine statistical significance. All error bars represent the SD (n = 3). *P ≤ .05; **P ≤ .01

To verify the impact of SFRP2 on SCAPs under the hypoxia and inflammatory conditions, we treated the cells with 10 ng/mL TNFα to mimic the inflammation condition and then culture SCAPs under hypoxia condition. Real‐time RT‐PCR result showed that SFRP2 was significantly decreased at 1, 2 and 4 hours after 10 ng/mL TNFα stimulation under the hypoxia condition (Figure 2A). Then, we found that the ALP activity was increased at 5 days in SCAPs‐HA‐SFRP2 compared with SCAPs‐Vector after 10 ng/mL TNFα stimulation under the hypoxia condition (Figure 2B). Then, Alizarin Red staining results demonstrated remarkably improvement of mineralization in SCAPs‐HA‐SFRP2 compared with SCAPs‐Vector after 10 ng/mL TNFα stimulation under the hypoxia condition (Figure 2C). Real‐time RT‐PCR results also displayed the osteo/odontogenic‐related genes DSPP, DMP1 and BSP were up‐regulated at 0, 7 and 14 days after osteo/odontogenic induction in SCAPs‐HA‐SFRP2 compared with control (Figure 2D‐F). Furthermore, real‐time RT‐PCR result displayed that the expression of OSX was also up‐regulated in SCAPs‐HA‐SFRP2 under the hypoxia and inflammation conditions (Figure 2G).

### 3.3 SFRP2 promoted paracrine potentials of SCAPs

SCAPs were cultured in normoxia condition, and then, the culture supernatants from SCAPs‐HA‐SFRP2 and SCAPs‐Vector cells were collected for proteomic analysis. From the proteomic data, 195 proteins were differently expressed, with 95 proteins up‐regulated, such as VEGFA, IGFBP5, IGFBP4, MMP1, MMP3, MMP13, CCL2, CCL7, CXCL5, CXCL12, CXCL6, CXCL8, CXCL3 and CXCL1, and 100 proteins were down‐regulated, such as ANKRD1, DES and GCLM in supernatants of SCAPs‐HA‐SFRP2 compared to the SCAPs‐Vector (Table S2).

### 3.4 SFRP2 enhanced the migration, chemotaxis and osteo/odontogenic ability of MSCs under hypoxia and inflammation conditions

**Figure 3:**
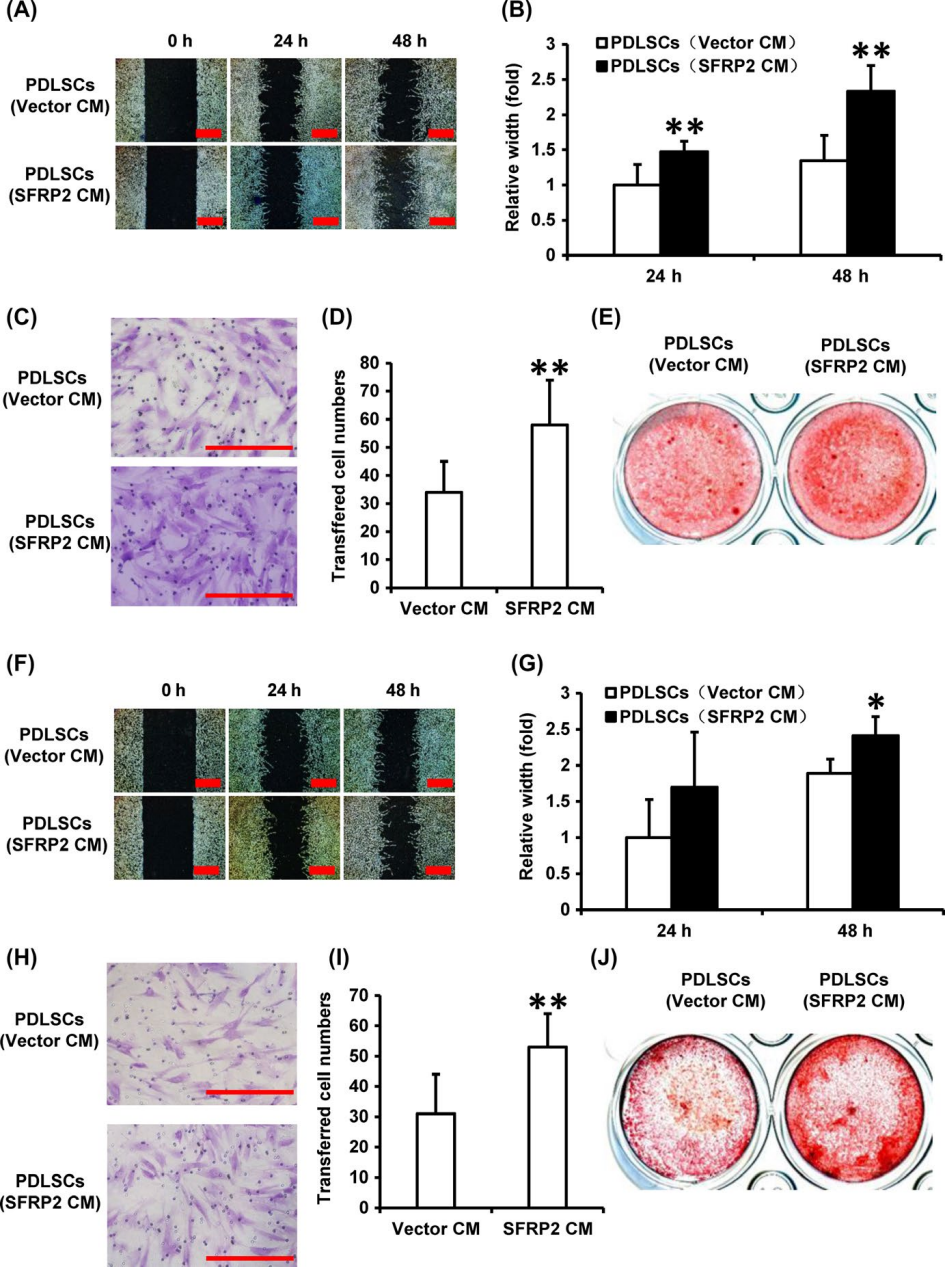
Supernatant of HA‐SFRP2‐infected SCAPs increased migration, chemotaxis and osteo/odontogenic potential of PDLSCs under hypoxia and inflammation conditions. PDLSCs were treated with culture medium supernatant of SCAPs‐Vector cells (Vector‐CM) or SCAPs‐HA‐SFRP2 cells (SFRP2‐CM). A‐E, PDLSCs were cultured under 3% hypoxia condition. The results of scratch migration assay (A) and quantitative analysis (B), the transwell chemotaxis assay (C) and quantitative analysis results (D), and Alizarin Red staining results (E). F‐J, PDLSCs were cultured under 3% hypoxia condition with 10 ng/mL TNFα simulation. The results of scratch migration assay (F) and quantitative analysis (G), the transwell chemotaxis assay (H) and quantitative analysis results (I), and Alizarin Red staining results (J). Student's *t* test was performed to determine statistical significance. All error bars represent the SD (n = 3). * *P* ≤ .05, **P ≤ .01. Scale bar: 100 μm

To determine the effect of SFRP2 on SCAPs secretion and function under hypoxia condition, PDLSCs were treated with the conditional medium (CM) collected from SCAPs‐HA‐SFRP2 or SCAPs‐Vector under hypoxia condition. The scratch migration assays showed a greater migration ability in SCAPs‐HA‐SFRP2‐CM‐treated PDLSCs than SCAPs‐Vector‐CM‐treated group at 24 hours and 48 hours (Figure 3A, 3B), and the transwell assay also revealed stronger chemotaxis ability in SCAPs‐HA‐SFRP2‐CM‐treated PDLSCs at 24h (Figure 3C, 3D). In vitro, Alizarin Red staining results showed increased mineralization in PDLSCs treated with SCAPs‐HA‐SFRP2‐CM than that of SCAPs‐Vector‐CM at 2 weeks (Figure 3E). Similarly, scratch migration assays, the transwell assay and Alizarin Red staining results showed that SCAPs‐HA‐SFRP2‐CM could also enhance migration, chemotaxis ability and mineralization in vitro in PDLSCs in hypoxia condition with TNFα stimulation (Figure 3F‐J).

Then, the conditional medium from SCAPs was also used to treat BMSCs. Scratch migration assays, the transwell assay and Alizarin Red staining results showed that SCAPs‐HA‐SFRP2‐CM could promote the ability of migration, chemotaxis and mineralization in vitro in BMSCs under hypoxia condition (Figure S4A‐E). And Scratch migration assays, the transwell assay and Alizarin Red staining results showed that SCAPs‐HA‐SFRP2‐CM could promote the ability of migration, chemotaxis and mineralization in vitro in BMSCs under hypoxia condition with 10 ng/mL TNFα treatment (Figure S4F‐J).

### 3.5 SFRP2 repressed NFκB Signalling through Inhibiting Wnt/β‐catenin pathway under hypoxia and inflammation conditions

**Figure 4:**
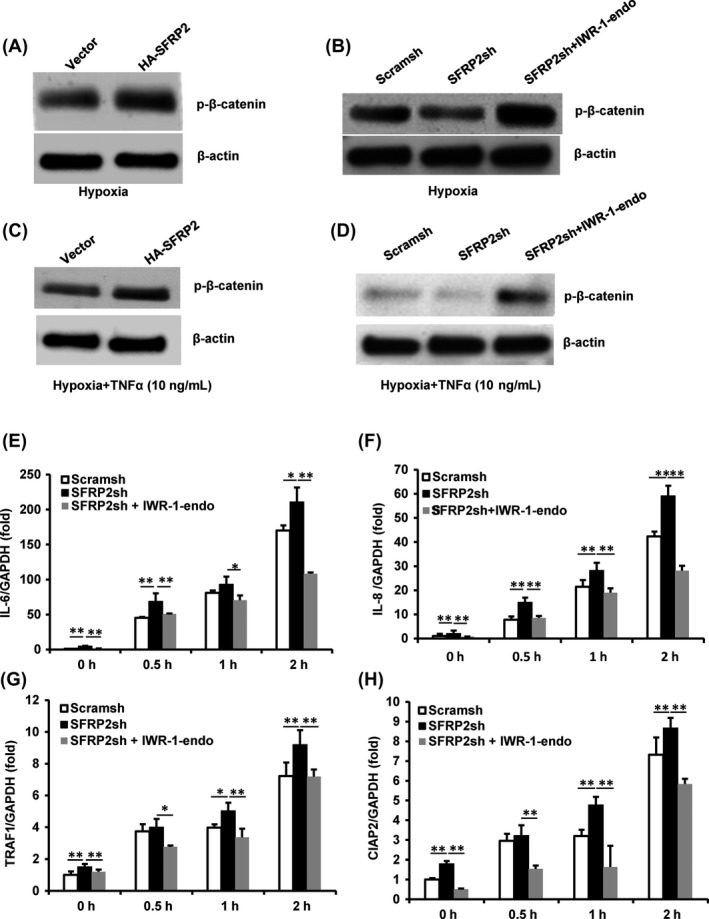
SFRP2 repressed NFκB signalling by inhibiting Wnt/β‐catenin pathway in SCAPs under hypoxia and inflammation conditions. A, Western blot results showed phosphorylation of β‐catenin in SCAPs‐HA‐SFRP2 and SCAPs‐Vector in 3% hypoxia condition. B, IWR‐1‐endo promote the decreased phosphorylation of β‐catenin in SFRP2‐depleted SCAPs in 3% hypoxia condition. C, Western blot results showed phosphorylation of β‐catenin in SCAPs‐HA‐SFRP2 and SCAPs‐Vector in 3% hypoxia condition with 10 ng/mL TNFα simulation for 24 h. D, Western blot results showed that IWR‐1‐endo increased the decreased phosphorylation of β‐catenin in SFRP2‐depleted SCAPs in 3% hypoxia condition with 10 ng/mL TNFα simulation for 24 h. E‐H, Real‐time RT‐PCR showed that IWR‐1‐endo reduced the expressions of IL‐6 (E), IL‐8 (F), TRAF1 (G) and CIAP2 (H) in SFRP2‐depleted SCAPs in 3% hypoxia condition with 10 ng/mL TNFα simulation. GAPDH was used as internal control in real‐time RT‐PCR. β‐actin was used as internal control in Western blot. One‐way ANOVA was performed to determine statistical significance. All error bars represent the SD (n = 3). *P ≤ .05; **P ≤ .01

Next, we explored whether SFRP2 enhanced the osteo/odontogenic differentiation via Wnt/β‐catenin signalling pathway in hypoxia and inflammation condition. Western blot results showed increased protein levels of phosphorylated β‐catenin (p‐β‐catenin) in SCAPs‐HA‐SFRP2 cells compared with SCAPs‐Vector group under hypoxia condition (Figure 4A). Western blot results also showed that depletion of SFRP2 decreased p‐β‐catenin level compared with control group (scramsh group), and Wnt/β‐catenin signalling inhibitor, IWR‐1‐endo, could restore the decreased p‐β‐catenin expression in SCAPs‐SFRP2sh group under hypoxia condition (Figure 4B). Then, Western blot results showed increased protein levels of p‐β‐catenin in SCAPs‐HA‐SFRP2 cells compared with SCAPs‐Vector group under hypoxia condition with 10 ng/mL TNFα stimulation (Figure 4C). We also found that SFRP2 depletion decreased p‐β‐catenin level compared with control group (scramsh group), and IWR‐1‐endo could restore the decreased p‐β‐catenin expression in SCAPs‐SFRP2sh group under hypoxia condition with 10 ng/mL TNFα stimulation (Figure 4D).

We also detected whether NFκB signalling pathway was involved in the process. Under hypoxia condition, we found increased levels of NFκB signalling pathway–related genes such as IL‐6, IL‐8, TRAF1 and CIAP2 in SCAPs after treated with 10ng/ml TNFα (Figure S5A‐D). Then under hypoxia condition, the real‐time RT‐PCR results demonstrated that IL‐6, IL‐8, TRAF1 and CIAP2 were also highly expressed in SCAPs‐SFRP2sh compared with control group after treated with 10 ng/mL TNFα; then, IWR‐1‐endo could repress the increased IL‐6, IL‐8, TRAF1 and CIAP2 expressions in SCAPs‐SFRP2sh (Figure 4E‐H). These results indicated that SFRP2 could repress NFκB signalling through inhibition of Wnt/β‐catenin signalling pathway under hypoxia and inflammation conditions in SCAPs.

### 3.6 KDM2A inhibited SFRP2 transcription by decreasing histone H3K4 and H3K36 methylation in SFRP2 promoter under hypoxia and inflammation conditions

**Figure 5:**
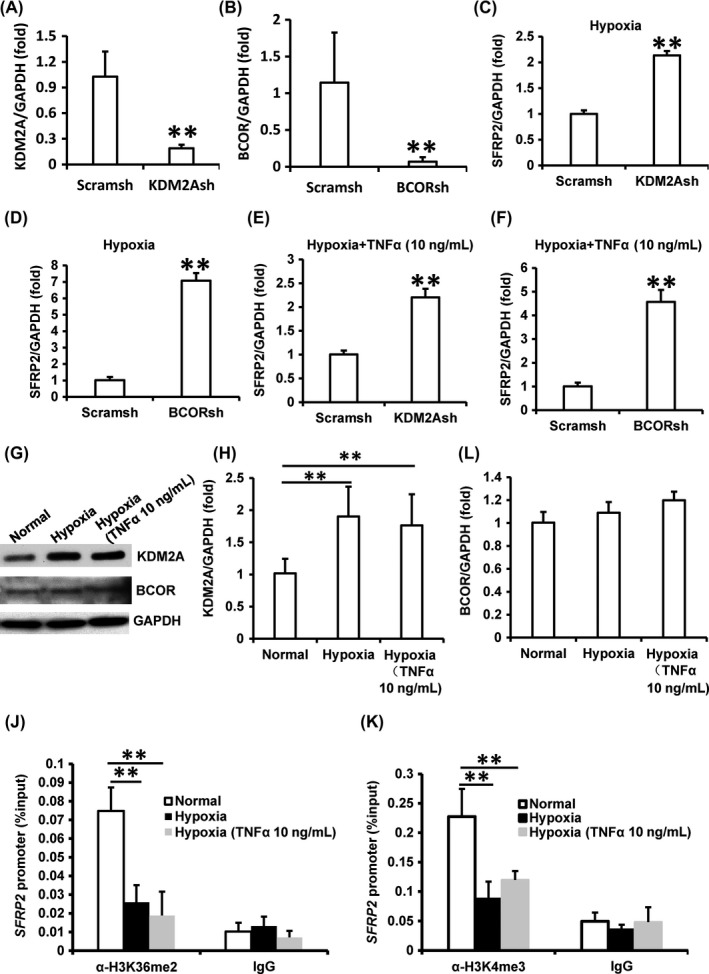
Hypoxia induced KDM2A and inhibited SFRP2 transcription by decreasing histone H3K4 and H3K36 methylation of the SFRP2 promoter in SCAPs. A‐F, SCAPs were cultured under 3% hypoxia condition. A, B, Knock‐down of KDM2A (A) and BCOR (B) in SCAPs was confirmed by real‐time RT‐PCR. C, D, Under hypoxia condition, real‐time RT‐PCR results showed that SFRP2 was up‐regulated in KDM2A‐ (C) or BCOR‐depleted SCAPs (D). E, F, Under hypoxia condition with 10 ng/mL TNFα simulation, real‐time RT‐PCR results showed that SFRP2 were up‐regulated in KDM2A‐ (E) or BCOR‐depleted SCAPs (F). G, Western blot showed expressions of KDM2A and BCOR in SCAPs under normoxia, hypoxia and hypoxia with 10 ng/mL TNFα simulation. H, I, Real‐time RT‐PCR showed expressions of KDM2A (H) and BCOR (I) in SCAPs under normoxia, hypoxia and hypoxia with 10 ng/mL TNFα simulation. J, K, ChIP assay showed decreased levels of H3K36me2 (J) and H3K4me3 (K) in the SFRP2 promoter under 3% hypoxia condition and 3% hypoxia with 10 ng/mL TNFα simulation. Student's *t* test or one‐way ANOVA was performed to determine statistical significance. All error bars represent the SD (n = 3). **P ≤ .01

In present study, we detected whether SFRP2 was downstream target gene of BCOR and KDM2A under predicted environment. First, real‐time RT‐PCR was used to confirm the knock‐down efficiency of KDM2A and BCOR in SCAPs (Figure 5A, 5B). Then, increased expression of SFRP2 was discovered in KDM2A knock‐down SCAPs or BCOR‐depleted SCAPs under hypoxia condition (Figure 5C, 5D). We also found that depletion of KDM2A or BCOR in SCAPs up‐regulated the expression of SFRP2 in hypoxia with 10ng/ml TNFα stimulation (Figure 5E, 5F). In the meantime, Western blot and real‐time RT‐PCR results demonstrated that KDM2A was up‐regulated in SCAPs under hypoxia condition with or without 10ng/ml TNFα stimulation compared with normoxia group, but there was no difference of BCOR expression among these three groups (Figure 5G‐I). Then, ChIP assay results showed that H3K36me2 and H3K4me3 levels were decreased in SFRP2 promoter in SCAPs under hypoxia condition with or without 10 ng/mL TNFα stimulation compared with normoxia condition (Figure 5J, K).

## 4 DISCUSSION

To reach the success of MSC‐mediated DPC regeneration requires conquering the impairment of inflammation and hypoxia in root canals. Thus, investigating the biological properties and mechanism of MSCs under hypoxia and inflammation conditions is important prerequisite for regulation of MSCs and enhancing MSC function.

In present study, we discovered that the expression of SFRP2 was decreased in pulpitis tissues. Then, we demonstrated the influence of the hypoxia and inflammation conditions on SFRP2 in MSCs. Our results confirmed that hypoxia and inflammation conditions inhibit the expression of SFRP2 in SCAPs. Our previous study showed the important role of SFRP2 on increasing the osteo/odontogenic differentiation of MSCs.^32^, ^33^ These indicated that under the conditions of hypoxia and inflammation, decreased SFRP2 might be a factor for the impaired differentiation properties of MSCs. Thus, we studied the function of SFRP2 under the conditions of hypoxia and inflammation. We found that SFRP2 enhanced the osteo/odontogenic differentiation of SCAPs under the hypoxia condition. Similarly, we also found that SFRP2 enhanced the osteo/odontogenic differentiation of SCAPs under the hypoxia and inflammation conditions. In addition, we found that SFRP2 also enhanced the migration and proliferation capacity of SCAPs under the hypoxia condition. But under the switch from hypoxia to normal conditions, the enhancement of cell proliferation by SFRP2 was diminished, and the promoted migration by SFRP2 also weakened. These indicated that the environment will affect the SFRP2 function. And the mechanism is unclear and needs further study.

In clinic, hypoxia and inflammation niche usually affect the functions of endogenous MSCs. So, promoting the functions of endogenous MSCs is also the key issue for the tissue regeneration. Multiple studies have elucidated that transplanted MSCs could modulate the function of endogenous MSCs and enhance the regeneration effects through paracrine action.^34^, ^35^, ^36^ SFRP2 was shown to be a key paracrine factor regulating myocardial survival and repair.^31^ In another research, the authors found that autocrine/paracrine SFRP2 induced cellular resistance to apoptosis.^37^ Therefore, we elucidated the paracrine function of SFRP2 on SCAPs. The proteomic data showed that SFRP2 enhanced SCAPs secreted more functional protein related to the osteo/odontogenic differentiation such as IGFBP5, IGFBP4 and MMP1, MMP3, MMP13, and some functional proteins related to cell homing, such as CXCL5, CXCL12, CXCL6, CXCL8, CXCL3 and CXCL1. Then, the condition medium from SCAPs was used to stimulate the PDLSCs and BMSCs, which were main endogenous MSCs around the root apex. Under hypoxia and inflammation conditions, conditional medium from SFRP2 over‐expressed SCAPs improved the migration, chemotaxis and mineralization abilities of PDLSCs and BMSCs, indicating that SFRP2 not only secreted more functional proteins and could recruit more endogenous MSCs taking part in tissue restoration, but also promoted the osteo/odontogenic differentiation potential of MSCs under hypoxia and inflammation conditions. Taken together, these findings indicated that SFRP2 was able to enhance the functions of exogenous and endogenous MSCs in the process of tissue regeneration under hypoxia and inflammation conditions.

We further investigate the mechanism of SFRP2 on the SCAPs under hypoxia and inflammation conditions. Wnt/β‐catenin components are expressed in the dental epithelium and mesenchyme during tooth development, and functional studies confirm the effects of Wnt signalling pathway on the regulation of tooth formation and tooth homeostasis.^38^, ^39^ SFRP2, a well‐known modulator of Wnt signalling, could prevent Wnt signalling by directly binding to Wnt molecule.^40^, ^41^ Previous study displayed inhibition function of SFRP2 on canonical Wnt signalling through enhancing phosphorylation expression level of β‐catenin.^33^ In this study, we confirmed the similar regulation relationship between SFRP2 and phosphorylated β‐catenin under hypoxia and inflammation conditions. More important, when treated with IWR‐1‐endo, a Wnt/β‐catenin signalling antagonist, the function modulation after SFRP2 depletion, was diminished, further indicating that SFRP2 function might be via inhibition of Wnt in MSCs.

NFκB signalling pathway is a key master of inflammation, and activation of NFκB signalling induces transcription of proinflammatory genes.^42^, ^43^ Previous studies also have defined the interaction between Wnt signalling and NFκB signalling pathway during inflammation. Some researchers showed an inflammation regulation function of Wnt/β‐catenin via regulation of NFκB signalling pathway.^44^, ^45^ Thus, we wondered whether the cross‐talk between Wnt signalling and NFκB signalling pathways participated in the SFRP2 regulation on MSCs. Then, we investigated whether the effect of SFRP2 in MSCs was related with NFκB signalling. Under hypoxia condition and with 10 ng/mL TNFα treatment, we found that depletion of SFRP2 enhanced the target genes of NFκB signalling including IL‐6, IL‐8, TRAF1 and CIAP2, and then, IWR‐1‐endo could repress this effect of SFRP2 depletion in SCAPs. These results indicated that SFRP2 could repress NFκB signalling through inhibition of Wnt/β‐catenin signalling pathway under hypoxia and inflammation conditions.

Moreover, previous study demonstrated that depletion of KDM2A increased H3K4me3 and H3K36me2 methylation via KDM2A‐BCOR protein complex at the SFRP2 promoter, which also caused the transcription de‐repression of SFRP2 in normoxia.^32^ Other investigation proved increasing of KDM2A and HIF‐1 in human cells under hypoxia condition, with HIF‐1 binding to the KDM2A promoter.^46^ In our study, we confirmed hypoxia and inflammation could increase the expression level of KDM2A in SCAPs, but did not affect the binding partner, BCOR expression. Furthermore, we found that hypoxia and inflammation conditions decreased the levels of H3K36me2 and H3K4me3 at the SFRP2 promoter, indicating that KDM2A regulated SFRP2 transcription through demethylation of H3K36me2 and H3K4me3 at the SFRP2 promoter under hypoxia and inflammation conditions. Taken together, we speculated that under hypoxia and inflammation conditions, HIF‐1 might increase the KDM2A expression and KDM2A formed more complex with BCOR, demethylated H3K36me2 and H3K4me3 at the SFRP2 promoter, and repressed the SFRP2 transcription. Thus, inhibition of KDM2A or KDM2A/BCOR complex might be other effective targets for improving the MSC function under hypoxia and inflammation conditions.

## 5 CONCLUSIONS

In present study, our discovery revealed that SFRP2 promoted the osteo/odontogenic differentiation and paracrine potentials of SCAPs under hypoxia and inflammation conditions. Mechanismly, hypoxia and inflammation could up‐regulate KDM2A expression in SCAPs, might form more complex with BCOR, and decreased H3K4me3 and H3K36me2 methylation at the SFRP2 promoter and inhibited SFRP2 transcription. Furthermore, SFRP2 inhibited the canonical Wnt/β‐catenin signalling and then repressed the NFκB signalling pathway. These findings provided the new function of SFRP2 and underlying mechanism on regulation of MSCs and identified some candidate targets for improving tissue regeneration under hypoxia and inflammation conditions.

## CONFLICT OF INTEREST

The authors declared that they have no competing interests.

## AUTHOR CONTRIBUTIONS

HY was responsible for collection and assembly of data analysis, interpretation and manuscript writing. GL was contributed to the collection and assembly of data analysis and interpretation. NH, XZ and YC were responsible for data collection. YC and ZF were responsible for conception, design, manuscript revising and confirmation, and financial support. All authors have read and approved the final version of the manuscript.

## Data Availability

Research data are not shared.
