# Supplementary figures and images for: Secreted frizzled‐related protein 2 promotes the osteo/odontogenic differentiation and paracrine potentials of stem cells from apical papilla under inflammation and hypoxia conditions
Source: Cell Prolif. 2019 Sep 30;53(1):e12694. doi: 10.1111/cpr.12694 (PMC6985663; doi:10.1111/cpr.12694)

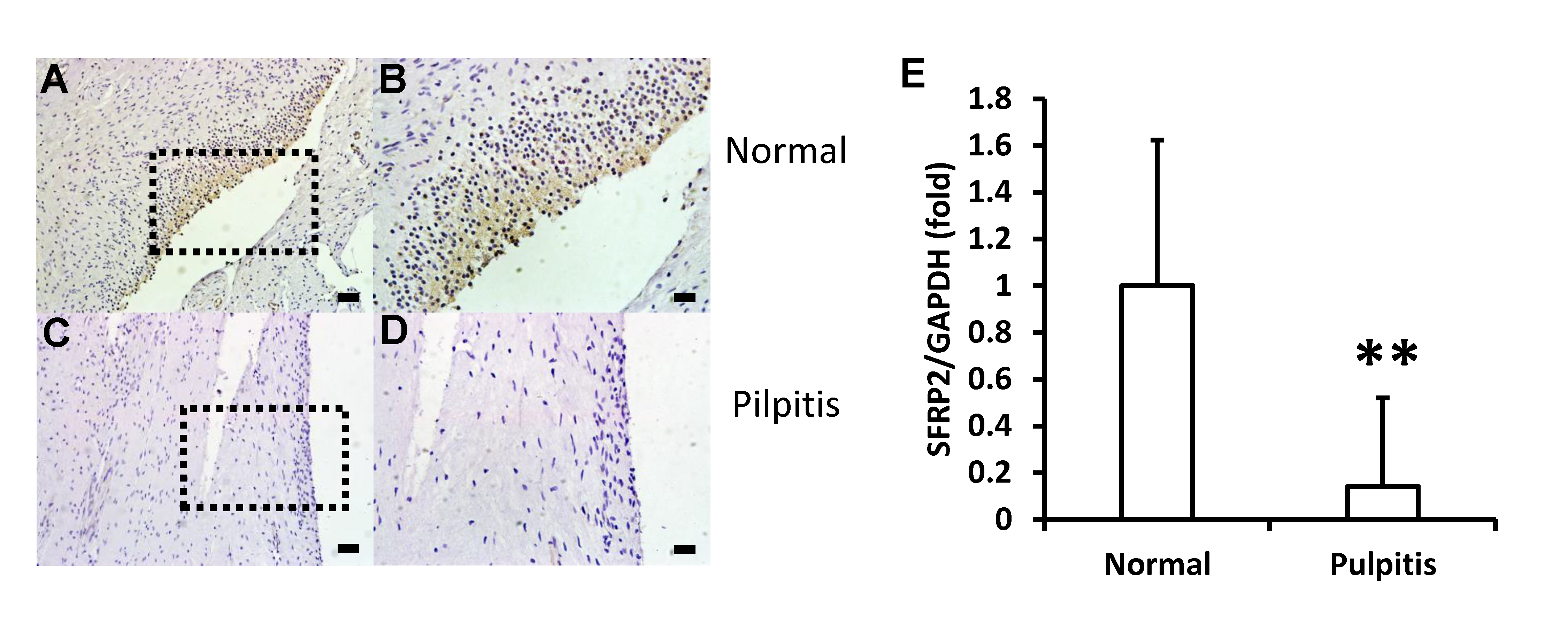

Supplement: Supplementary file 1 [file CPR-53-e12694-s001.tif]

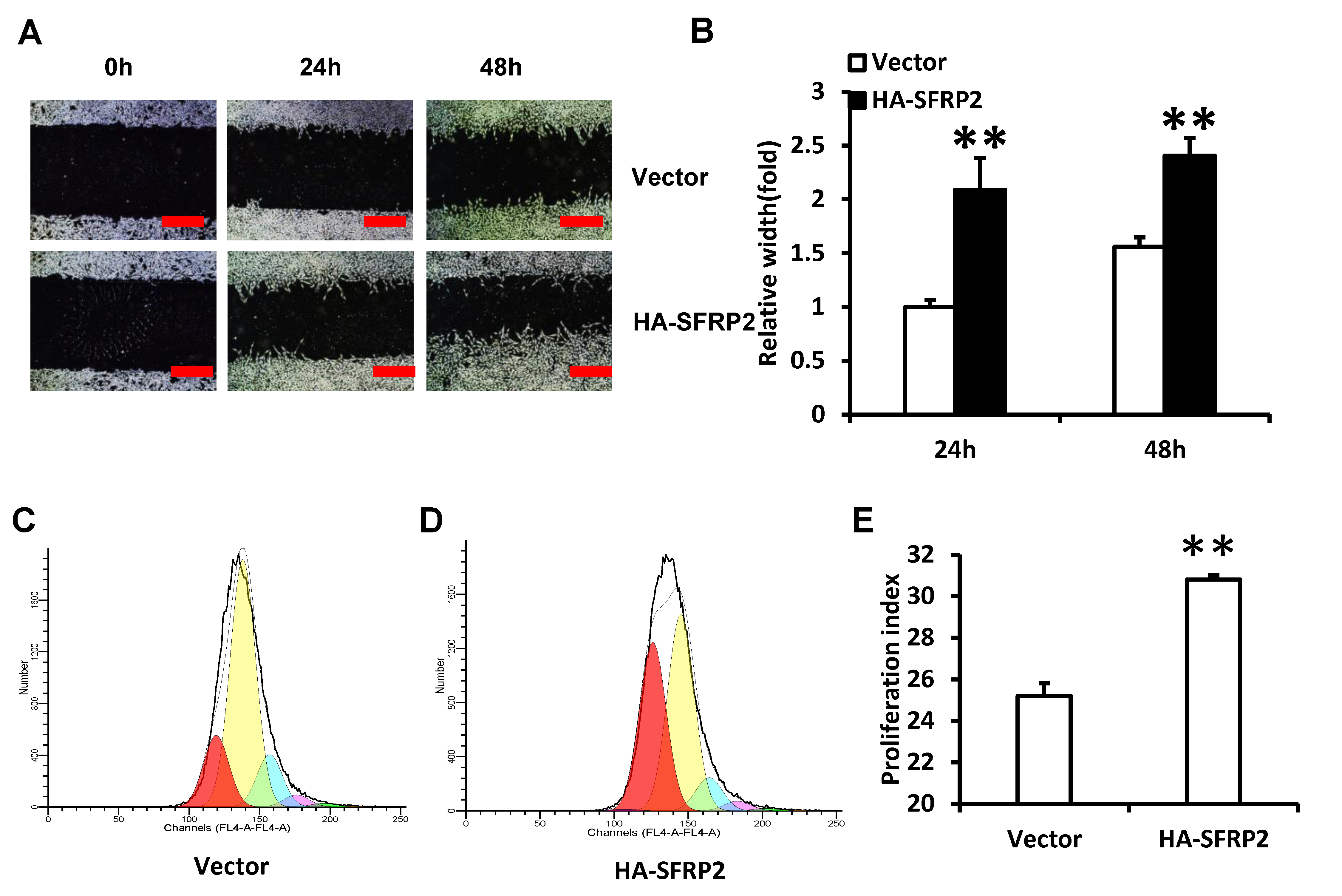

Supplement: Supplementary file 2 [file CPR-53-e12694-s002.tiff]

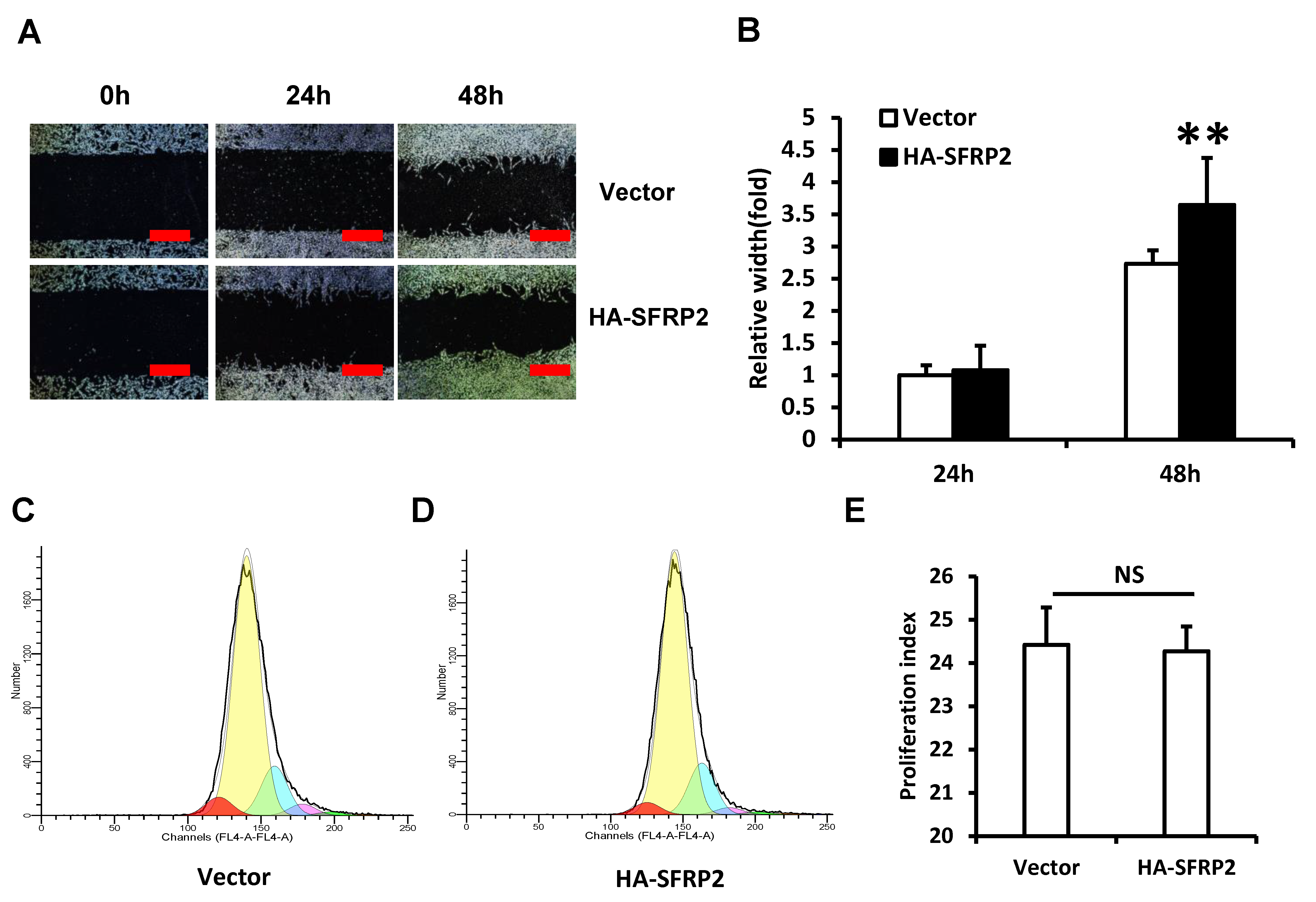

Supplement: Supplementary file 3 [file CPR-53-e12694-s003.tiff]

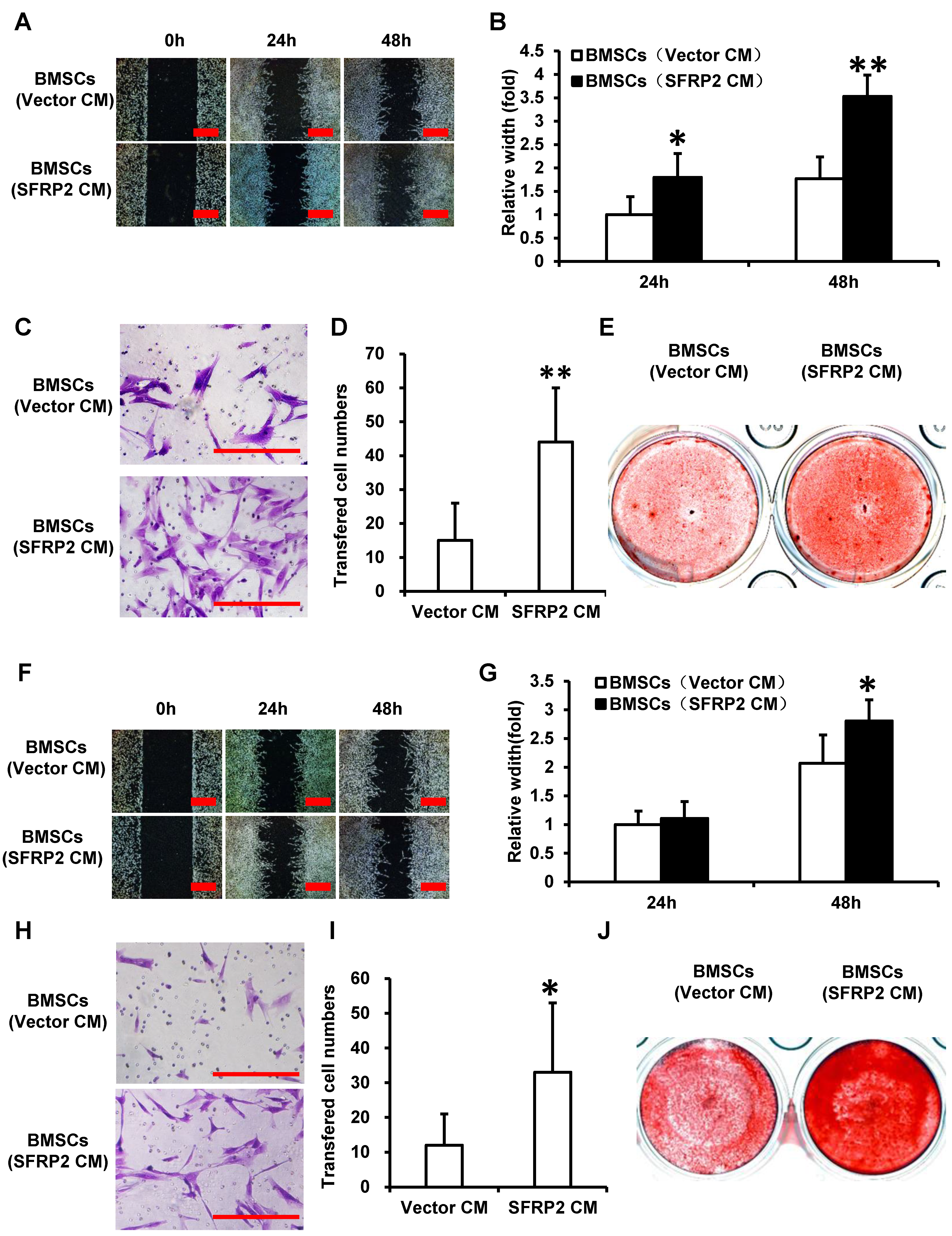

Supplement: Supplementary file 4 [file CPR-53-e12694-s004.tiff]
