# Supplementary material for: Secreted frizzled‐related protein 2 promotes the osteo/odontogenic differentiation and paracrine potentials of stem cells from apical papilla under inflammation and hypoxia conditions
Source: Cell Prolif. 2019 Sep 30;53(1):e12694. doi: 10.1111/cpr.12694 (PMC6985663; doi:10.1111/cpr.12694)
Supplement: Supplementary file 6 [file CPR-53-e12694-s006.docx]

**Supplementary Table 1. Primers sequences used in the real-time RT-PCR**

| **Gene Symbol** | **Primer Sequences (5’—3”)** |
| --- | --- |
| GAPDH-F | CGGACCAATACGACCAAATCCG |
| GAPDH-R | AGCCACATCGCTCAGACACC |
| KDM2A-F | CGGCCTCATCTTCTGACTGTAGCCG |
| KDM2A-R | CAGATGCACTTGCCTGTCCTTT |
| BSP-F | CAGGCCACGATATTATCTTTACA |
| BSP-R | CTCCTCTTCTTCCTCCTCCTC |
| OSX-F | CCTCCTCAGCTCACCTTCTC |
| OSX-R | GTTGGGAGCCCAAATAGAAA |
| BCOR-F | CATAGTGCTTGTGGAACTCCG |
| BCOR-R | GGACACAGCTCTCCTGTTGC |
| SFRP2-F | CGACATACAAAGCGTTTCCATTATGTCG |
| SFRP2-R | CACCGAGGAAGCTCCAAAGG |
| OPN-F | ATGATGGCCGAGGTGATAGT |
| OPN-R | ACCATTCAACTCCTCGCTTT |
| DSPP-F | CGACATAGGTCACAATGAGGATGTCG |
| DSPP-R | TTGCTTCCAGCTACTTGAGGTC |
| DMP1-F | CGTGGACAAAGAAGATAGCAACTCCACG |
| DMP1-R | TTCCGGCTCTCTATCTCAATGTTT |
| IL8-F | ACAGCGGATCGTAAAGGCAA |
| IL8-R | CGGGAGCGGTTCTGAGATTT |
| IL6-F | CGCAACAACTCATCTCATTCTGCG |
| IL6-R | CATGCTACATTTGCCGAAGAGC |
| TRAF1-F | CGGTGCTCTTGATCCCTACTCACCG |
| TRAF1-R | GAATGGCTGCATCTCATGCTCT |
| CIAP2-F | CAACAGATCTGGCAAAAGCA |
| CIAP2-R | ATTTTCCACCACAGGCAAAG |
