# Supplementary material for: Secreted frizzled‐related protein 2 promotes the osteo/odontogenic differentiation and paracrine potentials of stem cells from apical papilla under inflammation and hypoxia conditions
Source: Cell Prolif. 2019 Sep 30;53(1):e12694. doi: 10.1111/cpr.12694 (PMC6985663; doi:10.1111/cpr.12694)
Supplement: Supplementary file 7 [file CPR-53-e12694-s007.docx]

**Supplementary Table 2.proteomic analysis result of culture supernatants from SCAPs-HA-SFRP2 and SCAPs-Vector cells.**

| Gene names | Regulation | Ratio（SCAP-HA-SFRP2/ SCAP-Vector） |
| --- | --- | --- |
| SFRP2 | UP | 4.302694 |
| FAM213B | UP | 2.057315 |
| CCL7 | UP | 1.972697 |
| CXCL5 | UP | 1.918735 |
| TSKU | UP | 1.777803 |
| PLAT | UP | 1.705919 |
| TNFAIP6 | UP | 1.689595 |
| PAG1 | UP | 1.647997 |
| TFPI2 | UP | 1.640161 |
| STC1 | UP | 1.617468 |
| HIST1H4A; | UP | 1.612421 |
| MMP13 | UP | 1.530833 |
| VEGFA | UP | 1.522853 |
| MMP1 | UP | 1.51854 |
| TRMT1 | UP | 1.512244 |
| SH3PXD2B | UP | 1.492348 |
| L3HYPDH | UP | 1.490029 |
| SCG2 | UP | 1.484951 |
| CXCL12 | UP | 1.482558 |
| MMP3 | UP | 1.460468 |
| OLFML2B | UP | 1.456738 |
| IPO4 | UP | 1.456705 |
| TXNL4A | UP | 1.454949 |
| VAPB | UP | 1.454861 |
| HLA-B | UP | 1.447089 |
| CXCL6 | UP | 1.430763 |
| CCL2 | UP | 1.424003 |
| HDDC3 | UP | 1.413028 |
| HIST1H2AB; | UP | 1.403919 |
| ST8SIA4 | UP | 1.398409 |
| PRAMEF1 | UP | 1.39533 |
| IGFBP5 | UP | 1.394819 |
| IGDCC4 | UP | 1.394751 |
| SOD2 | UP | 1.392531 |
| ARMC5 | UP | 1.385617 |
| WNT5A | UP | 1.383881 |
| CDKN2AIP | UP | 1.374419 |
| LSM12 | UP | 1.367948 |
| AP1S1 | UP | 1.365829 |
| GALM | UP | 1.360429 |
| SPON1 | UP | 1.359215 |
| SFRP1 | UP | 1.358633 |
| ITM2B | UP | 1.352564 |
| HIST1H2BK | UP | 1.350155 |
| HIST1H2BJ | UP | 1.332915 |
| VCAN | UP | 1.330471 |
| NUFIP2 | UP | 1.32507 |
| NTNG1 | UP | 1.32339 |
| HIST1H1E | UP | 1.320872 |
| CLASP2 | UP | 1.319179 |
| HGF | UP | 1.314046 |
| EFEMP1 | UP | 1.313365 |
| SMPDL3A | UP | 1.308953 |
| CXCL8 | UP | 1.304448 |
| HIST1H1C | UP | 1.298325 |
| NRCAM | UP | 1.29534 |
| C3 | UP | 1.284546 |
| CXCL3 | UP | 1.283485 |
| IL6 | UP | 1.276165 |
| LEMD2 | UP | 1.272921 |
| IGFBP4 | UP | 1.272549 |
| KIRREL | UP | 1.272319 |
| TNFRSF21 | UP | 1.267161 |
| PTPRG | UP | 1.264881 |
| HIST1H1B | UP | 1.262162 |
| RAD1 | UP | 1.258459 |
| UBXN7 | UP | 1.253519 |
| STAT1 | UP | 1.247579 |
| SF3B6 | UP | 1.242288 |
| LAMTOR5 | UP | 1.241797 |
| CHMP2A | UP | 1.241177 |
| SECTM1 | UP | 1.234339 |
| PF4 | UP | 1.232416 |
| PPP4C | UP | 1.232114 |
| C1S | UP | 1.230407 |
| IRF3 | UP | 1.229466 |
| RNASE4 | UP | 1.225565 |
| SATB2 | UP | 1.225023 |
| MRTO4 | UP | 1.224943 |
| DNAJB6 | UP | 1.221624 |
| TRIM16L | UP | 1.218345 |
| MEGF10 | UP | 1.217331 |
| ISG15 | UP | 1.215386 |
| SEC16A | UP | 1.214101 |
| ATXN2 | UP | 1.21223 |
| DEK | UP | 1.211763 |
| SULF2 | UP | 1.211359 |
| WIZ | UP | 1.210668 |
| TGFB2 | UP | 1.207996 |
| RPS27 | UP | 1.207975 |
| SPOCK1 | UP | 1.206592 |
| CXCL1 | UP | 1.206278 |
| HIST2H2AB | UP | 1.203938 |
| JPT1 | UP | 1.202438 |
| ANXA4 | UP | 1.202044 |
| CAT | DOWN | 0.833009 |
| LEMD3 | DOWN | 0.832737 |
| RPL27 | DOWN | 0.832422 |
| PAMR1 | DOWN | 0.832309 |
| PSMF1 | DOWN | 0.831088 |
| GJA1 | DOWN | 0.831026 |
| COL12A1 | DOWN | 0.830526 |
| LEO1 | DOWN | 0.830297 |
| COL1A1 | DOWN | 0.829447 |
| C1orf122 | DOWN | 0.829213 |
| EXOSC10 | DOWN | 0.829086 |
| CST4 | DOWN | 0.828652 |
| CAMK2G | DOWN | 0.828646 |
| SMG1 | DOWN | 0.826827 |
| ABCF1 | DOWN | 0.826635 |
| IGFBP7 | DOWN | 0.821137 |
| UBE4B | DOWN | 0.820986 |
| OLFML3 | DOWN | 0.819405 |
| ADAM19 | DOWN | 0.81916 |
| LTBP2 | DOWN | 0.818804 |
| GBP1 | DOWN | 0.818012 |
| PPM1F | DOWN | 0.817841 |
| VTN | DOWN | 0.817567 |
| COL8A1 | DOWN | 0.81632 |
| ARIH2 | DOWN | 0.815504 |
| UROS | DOWN | 0.814492 |
| PEF1 | DOWN | 0.809714 |
| MRE11 | DOWN | 0.808726 |
| SRSF9 | DOWN | 0.807836 |
| IGFBP3 | DOWN | 0.806819 |
| AP1M1 | DOWN | 0.806727 |
| GLUL | DOWN | 0.805553 |
| IGF2 | DOWN | 0.804991 |
| KRT19 | DOWN | 0.802377 |
| KDELC2 | DOWN | 0.799515 |
| PPIE | DOWN | 0.797996 |
| ADAMTS7 | DOWN | 0.797817 |
| GMPPA | DOWN | 0.797398 |
| COL4A4 | DOWN | 0.79673 |
| INTS3 | DOWN | 0.794867 |
| POSTN | DOWN | 0.792878 |
| TSPAN6 | DOWN | 0.791882 |
| APOC3 | DOWN | 0.791291 |
| EDIL3 | DOWN | 0.790339 |
| HBB | DOWN | 0.790201 |
| COL4A6 | DOWN | 0.789378 |
| FLT1 | DOWN | 0.788768 |
| SEMA7A | DOWN | 0.786848 |
| SERPINE1 | DOWN | 0.786824 |
| PLCG1 | DOWN | 0.785983 |
| TAGLN | DOWN | 0.784955 |
| ACSS2 | DOWN | 0.784716 |
| HAPLN1 | DOWN | 0.781184 |
| SPARC | DOWN | 0.779354 |
| ST6GAL1 | DOWN | 0.775557 |
| THBS1 | DOWN | 0.775016 |
| A2M | DOWN | 0.769484 |
| CTGF | DOWN | 0.769204 |
| QPCT | DOWN | 0.768714 |
| PTX3 | DOWN | 0.766977 |
| VPS13C | DOWN | 0.766514 |
| BGN | DOWN | 0.762841 |
| SERPINE2 | DOWN | 0.755949 |
| FLOT2 | DOWN | 0.755363 |
| SVEP1 | DOWN | 0.749217 |
| NEDD4 | DOWN | 0.742033 |
| OMD | DOWN | 0.737972 |
| EXOSC9 | DOWN | 0.736031 |
| YPEL5 | DOWN | 0.733274 |
| CCBE1 | DOWN | 0.730497 |
| ETFA | DOWN | 0.726929 |
| MAP1S | DOWN | 0.725461 |
| STC2 | DOWN | 0.716291 |
| PDS5A | DOWN | 0.715355 |
| INTS13 | DOWN | 0.714584 |
| ITGBL1 | DOWN | 0.713322 |
| FBLN5 | DOWN | 0.712044 |
| COL15A1 | DOWN | 0.711362 |
| RPS6 | DOWN | 0.710028 |
| RPL30 | DOWN | 0.707907 |
| KPNA4 | DOWN | 0.699759 |
| NCOR1 | DOWN | 0.696812 |
| NFU1 | DOWN | 0.695459 |
| FBN2 | DOWN | 0.694601 |
| MMP11 | DOWN | 0.693 |
| PENK | DOWN | 0.687915 |
| VPS37A | DOWN | 0.681424 |
| GCLM | DOWN | 0.667522 |
| IGF2BP2 | DOWN | 0.659242 |
| GFRA1 | DOWN | 0.643514 |
| ADAMTS1 | DOWN | 0.629622 |
| PARP4 | DOWN | 0.623369 |
| GAS6 | DOWN | 0.614885 |
| CARD19 | DOWN | 0.599191 |
| RTF1 | DOWN | 0.59352 |
| HS3ST3B1 | DOWN | 0.517524 |
| MT1H | DOWN | 0.486203 |
| FUT11 | DOWN | 0.387935 |
| ANKRD1 | DOWN | 0.308532 |
| DES | DOWN | 0.263517 |
